# The Application of Fractal Transform and Entropy for Improving Fault Tolerance and Load Balancing in Grid Computing Environments

**DOI:** 10.3390/e22121410

**Published:** 2020-12-15

**Authors:** Murad B. Khorsheed, Qasim M. Zainel, Oday A. Hassen, Saad M. Darwish

**Affiliations:** 1 College of Administration & Economics, University of Kirkuk, Kirkuk 36001, Iraq; muradbahram@uokirkuk.edu.iq; 2 College of Physical Education and Sports Sciences, University of Kirkuk, Kirkuk 36001, Iraq; qasim@uokirkuk.edu.iq; 3 Computer Science and Information Technology, University of Wasit, Al Kut 52001, Iraq; odayali@uowasit.edu.iq; 4 Department of Information Technology, Institute of Graduate Studies and Research, Alexandria University, 163 Horreya Avenue, El–Shatby, Alexandria 21526, Egypt

**Keywords:** fractal transform, entropy estimation, grid computing, load balance, fault tolerance, optimization

## Abstract

This paper applies the entropy-based fractal indexing scheme that enables the grid environment for fast indexing and querying. It addresses the issue of fault tolerance and load balancing-based fractal management to make computational grids more effective and reliable. A fractal dimension of a cloud of points gives an estimate of the intrinsic dimensionality of the data in that space. The main drawback of this technique is the long computing time. The main contribution of the suggested work is to investigate the effect of fractal transform by adding R-tree index structure-based entropy to existing grid computing models to obtain a balanced infrastructure with minimal fault. In this regard, the presented work is going to extend the commonly scheduling algorithms that are built based on the physical grid structure to a reduced logical network. The objective of this logical network is to reduce the searching in the grid paths according to arrival time rate and path’s bandwidth with respect to load balance and fault tolerance, respectively. Furthermore, an optimization searching technique is utilized to enhance the grid performance by investigating the optimum number of nodes extracted from the logical grid. The experimental results indicated that the proposed model has better execution time, throughput, makespan, latency, load balancing, and success rate.

## 1. Introduction

Fractals are of a rough or fragmented geometric shape that can be subdivided into parts, each of which is a reduced copy of the whole. They are crinkly objects that defy conventional measures, such as length, and are most often characterized by their fractal dimension. They are mathematical sets with a high degree of geometrical complexity that can model many natural phenomena. Almost all natural objects can be observed as fractals [1]. Concepts from fractal theory have been applied to several tasks in data mining and data analysis, such as selectivity estimation, clustering, time series forecasting, correlation detection, and data distribution analysis [2]. Fractal tree properties include log(N) arrays, one array for each power of two, fractal tree indexes can use 1/100th the power of B-trees, and fractal tree indexes ride the right technology trends. In the future, all storage systems will use fractal tree indexes [3,4,5,6,7].

Grid technology has arisen as modern, high-performance-oriented, large-scale distributed networking. Grid computing is adopted in different fields, from university science to government application. The aim of grid computing is to establish the appearance of a simple yet efficient self-managing virtual machine from a wide set of linked heterogeneous networks sharing different resource combinations [8]. Grid management information is characterized as the process of defining specifications, matching resources with applications, resource allocation, scheduling, and tracking system resources over time so that grid applications can operate as efficiently as possible [9]. Resource exploration is resource management’s first step. The next phase is scheduling and monitoring. The scheduling mechanism leads the work to sufficient resources and the tracking mechanism tracks resources. The highly loaded resources will act as a server of the task and the loosely loaded resources will act as a receiver of the task. The task can move from a highly loaded node to a loaded server. Resources are complex in design, so power load changes with changing grid configuration. Due to some reasons, while processing any task, a defective device may trigger some harm. As a consequence, load balancing and task fault tolerance in a grid setting will greatly affect grid performance [10].

Load balancing’s key objective is to have a distributed low-cost scheme that spreads the demand over all processors. Powerful and effective algorithms that have been proposed are critical to improving global grid power performance. Load handling systems may be categorized as clustered (centralized) or distributed (decentralized) and dynamic or static, as well as periodic or non-periodic [10]. Owing to the complexity and complex hierarchical design (heterogeneity) of grid networks, task scheduling in the grid systems is more complicated than in conventional distributed computing systems [11]. This issue in grid design is solved by distributing load in a grid through neglecting the overhead contact in gathering load details. A centralized paradigm for a homogenous grid was implemented as a series of clusters through a tree network for grid operators who are capable of controlling a complex pool of computing machines or processors.

With respect to the principle of fault tolerance, the key challenges confronting grid structures are viability (feasibility), stability (reliability), usability (scalability), and connectivity [12]. A fault may be accepted depending on its actions (e.g., loss stop system) or even the manner in which it happens (network faults). There are several common fault tolerance approaches in the research, including repetition, scan-pointing, and scheduling/redundancy [13]. Replication is the knowledge exchange mechanism that maintains continuity between duplicate services. Scan-pointing is the saving mechanism from full mission execution. On the other side, scheduling/redundancy could be used for task scheduling and fault tolerance in distributed network dependence in resource or system upgrades and is used to address the downside of fill-pointing in cloud systems.

### Motivation and Contribution

Job and resource scheduling has been one of grid computing’s main study fields. It is therefore very challenging to identify an effective resource distribution approach that reduces the system load due to tasks, machines, and network connectivity. Even though many heuristics algorithms are available for scheduling the jobs, they are not effectively used because these algorithms do not consider both the system and job requirements. If they are considered, the performance of the grid system can be improved. This is the main motivating factor behind this research work that seeks to develop an efficient scheduling method for a grid environment. To achieve this goal, a new approach that integrates both fractal transform and entropy for grid network reduction in a unified framework is suggested to handle fault tolerance and load balance. This model depends on the fractal transform that enhances the tree model structure of the grid computing environment to enhance the network performance parameters affected by fault tolerance and load balance equally.

The following parts are structured as follows. Section 2 provides a summary of similar work. Section 3 describes the suggested model. Section 4 offers the results of numerical tests to confirm the research model. Finally, Section 5 draws conclusions and discusses them.

## 2. Related Work 

Cao (2005) [14] suggested a grid load balancing approach utilizing artificial intelligence to accomplish efficient workload and resource management. A mixture of smart agents and multi-agent methods is implemented in local grid resource scheduling and global grid load balancing. Each agent represents a local grid resource that uses predictive device output data with incremental heuristic algorithms to maintain local load balance throughout different servers. Yagoubi and Slimani (2006) [15] presented a layered balancing algorithm focused on tree representation. This model turns every grid design into a four-level, specific tree. It generates a two-level sub-tree for each site. This tree’s leaves reflect a site’s computational components, and the root represents a site-related virtual node. These sub-trees, referring to cluster locations, are clustered together to create a three-level sub-tree. Finally, such sub-trees are clustered together, creating a four-level tree called the generic load balancing model.

Yan (2009) [16] offered a hybrid network load policy integrating static or dynamic task scheduling architectures. The static load balancing policy is used to choose an appropriate and convenient node set. As a node shows potential failure to continue supplying services, the complex load balancing strategy can decide whether or not the node involved is successful in providing load distribution. In a short period, the device can receive a new substitute node to preserve system efficiency. Hao et al. (2012) [17] recommended a load structured Min-Min algorithm, mainly implemented to reduce start making span and increase resource utilization in the heterogeneous network. It is introduced in two steps. The Min-Min method is adopted in the first phase to schedule tasks. In the second stage, activities in crowded resources are postponed to effectively use underused resources.

The authors in [18] introduced an augmenting hierarchical load balancing. The possibility of deviation of mean system load from cluster load is calculated as well as checked for containment within a specified range from 0 to 1. The best tools are allocated to jobs by matching the predicted work computing capacity with cluster average computer power. The authors provided a grouping-based schedule of data storage for fine-grained work. It groups fine-grained jobs to form coarse-grained jobs based on resource processing capability and grouped job processing requirements. The analysts round about an asset and calculate the MIPS product and granularity time used to estimate the overall number of jobs that could be completed within the same specified timeframe. Then they select the resource with the required amount of waiting jobs [19].

Balasangameshwara (2012) [20] addressed numerous fault recovery processes, including checkpointing, replication, and rescheduling. Having to take checkpoints is the process of regularly saving the status of a permanent storage process. This makes a process that fails to restart from the point of last saving its state or checkpoint on another resource. Replication involves maintaining a sufficient amount of replicas or copies of parallel systems on various resources such that at least one copy succeeds. The rescheduling procedure finds various resources to reschedule failures.

The authors in [21] built a grid fault-tolerant scheduling method to plan backups and minimize job response time. In this approach, directed acyclic graphs model jobs. They schedule jobs with delays to avoid execution failures even with the appearance of processor faults. Initially, a communication framework is designed that defines when contact between such a backup and successor backups is needed. Then any processor malfunction will initiate the backup. This minimizes reply time and expense. The authors in [22] introduced a fault tolerance checkpointing mechanism. The checkpointing method regularly saves the condition of a process operating on a computational resource so it can restart on another resource in case of resource loss. If any resource faults occur, it invokes the required replicas to meet user application capacity needs. Lee et al. (2011) [23] developed a bi-criteria task scheduling that considers users’ satisfaction and fault tolerance. It concentrates on a pro-active fault-tolerant mechanism that considers resource failure history while scheduling jobs. It considers user date and job fulfillment period at all tools, and measures fitness value. Then jobs are scheduled depending on fitness value.

Both these heuristic schedulers discussed here have benefits and even certain drawbacks. The duplicitous load balancing does not regard the planned execution period and therefore is weak. The minimum execution time (MET) heuristic algorithm does not consider the completion of jobs, resulting in extreme load imbalance. Minimum completion time (MCT) often implies a low makespan. The Max-Min heuristic procedure is stronger than all these algorithms, but only for the shortest jobs. Of all these evolutionary methods discussed, the Min-Min algorithm is quick, fast, and plays better without being given user satisfaction, considering machine efficiency by reduced makespan. The application demand aware approach works best when taking into account user satisfaction. Many schedulers concentrate on user deadline and task scheduling separately, but no scheduler considers user deadline and resource load. Therefore, there is a wide potential for associated data focusing on both factors. The need to expand the relevant work is the scope of this article, improving both load balancing and fault tolerance with a new selected framework focused on a theory of fractal transformation and entropy.

The current methods address both load balancing and fault tolerance in grid environments separately [24,25,26,27,28,29,30,31,32,33]. The method proposed in this paper deals with each of them within one framework by combining some classical methods based on fractal transform and entropy computation for reducing the complexity of data to obtain an optimal method for rendering the grid structure. The study proposes scheduling algorithms that resolve numerous issues, like customer satisfaction, data aggregation, and fault tolerance, by considering criteria such as failure rate, load status, user deadline, and resource usage of scheduling services. In fact, for the first time, we have used the fractal transform and entropy in order to reduce the complexity of the grid.

## 3. Materials and Method 

**Figure 1:**
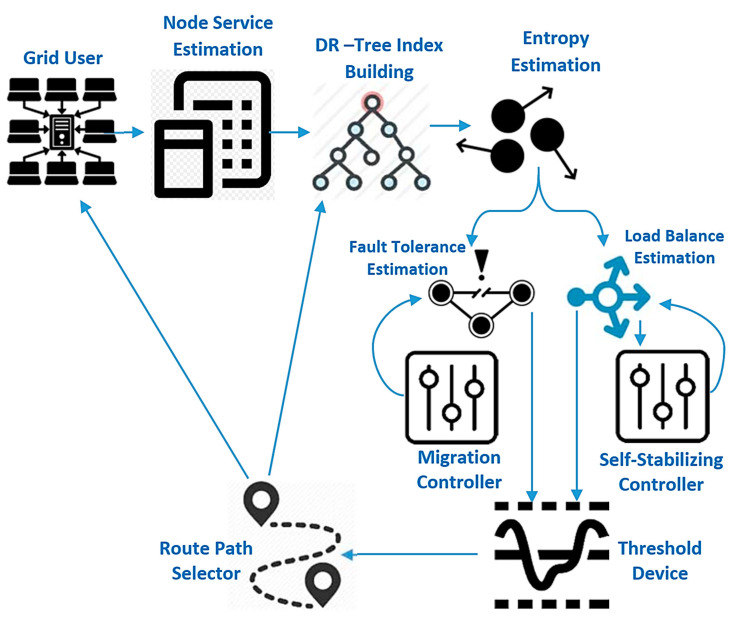
The proposed model for fault tolerance and load balance based on fractal transform.

Figure 1 shows the complete proposed model for improving fault tolerance and load balance based on the fractal transform. The first step is to estimate grid computing service (GCS) parameters, including cost, job queue, task schedule, cluster size, grid size, and the number of resources, then mapping this grid structure into a distributed R-tree index structure enhanced by the entropy method to reduce the completion time of the decision-maker. Finally, the threshold machine is used to choose the route path based on load balance as well as the fault tolerance device. The migrant controller is used to increase fault tolerance and self-stabilizing control is used to increase cumulative state load balancing. Each consumer submits their computational and hardware specifications to the GCS. The GCS can respond by submitting the results when the job execution is done.

In the GCS, jobs pass through four stages, which can be summarized as follows: (1) task submission phase: grid users can submit their jobs through the available web browsers. This makes the work submission process simple and open to any number of clients. (2) Task allocation phase: when the GCS receives work, it searches for the resources available (computers or processors) and assigns the necessary resources for the task. (3) Task execution phase: until the services available are committed to the assignment, the task is planned to be carried out on that computer location. (4) Results collection phase: when the jobs have been done, the GCS will alert the users of the results of their work. The proposed model would examine network parameters; GCS estimates by evaluating the top-down view of the grid model, which includes the local grid manager (LGM), site manager (SM), and processing elements (PEs) [34]. In this hierarchy, incorporating or deleting SMs or PEs becomes very versatile and tends to render the proposed grid computing service model open and scalable. 

The LGM’s task is to assess information regarding active resources depends on its SMs. LGMs also participate in grid-specific tasks and load balancing. New SMs can enter the GCS by sending a message to join the nearest LGM parent. Each SM is capable of managing a dynamically configured pool of processing units (computers as well as processors) (i.e., processing elements can join the pool anytime). A new computing element should be registered within the SM. The SM’s function is to gather information regarding active input nodes in its pool. The details gathered contains CPU speed and other hardware measurements. Any SM is also responsible for allocating incoming jobs to any processor core in its pool using a defined load balancing. Any public or private PC or workstation can enter the grid system by signing up with any SM and offering grid users their computing resources. As a computational unit enters the grid, it begins the GCS framework that will submit any details about its capabilities, such as processor power, to the SM. Any LGM is the grid model’s web server. Using the web browser, customers assign their computer science jobs to an associated LGM. According to the load balancing relevant data, the LGM will pass the published jobs to a suitable SM. The SM, in turn, distributes these computing jobs according to available site high availability information to a selected execution processing element. 

### 3.1. Building Distributed R-Tree (DR-Tree) Using Fractal Transform 

The self-similarity property can define a fractal, i.e., an object with roughly the same features over a wide range of scales [1]. Accordingly, a real dataset exhibiting fractal actions is exactly or empirically self-similar, so parts of any data size present the same whole dataset characteristics. The fractal dimension [2,3,4] is especially useful for data analysis from fractal theory as it offers an estimate of its inherent dimension *D* of datasets. The underlying dimension provides the complexity of the entity described by the data independent of the dimension *E* of the domain in which it is embedded. That is, *D* measures real data’s non-uniformity behavior. For example, a set of points representing a plane immersed in some kind of a 3-dimensional space (*E* = 3) has independent attributes and one third associated with the other, resulting in *D* = 2. Correlation recursive dimension $D_{2}$ can determine the spatial pattern of real datasets. The box-counting technique defines $D_{2}$ as an efficient tool for measuring the spatial pattern of datasets embedded throughout *E*-dimensional spaces:

(1) $$D_{2}\equiv \frac{\partial \;log\left(\sum_{i}C_{r,i}^{2}\right)}{\partial \log\left(r\right)}\;\;\;\;\;r\in \left[r_{1},r_{2}\right]$$

where *r* is the cell side in a (hyper) spherical grid separating the dataset’s address space, and $C_{r.i}$ is the count of points in the *i*th cell. Thus, *D*_2_ could be a valuable method for estimating a real dataset’s intrinsic parameter *D* with feasible computing expenses.

First, the tree model of grid computing nodes is converted into a DR-tree to reduce the complexity of the grid computing network due to the similarity properties with the tolerated and balanced R-tree index structure that can be run in logarithmic time. The idea is to promote the deferred splitting strategy in R-trees. This is achieved by seeking an R-tree node order. This ordering must be “nice” in that it must group “same” information nodes with whose representation can be contained in a rectangle of compact spaces. Each node has a very well-defined collection of sibling nodes providing the ordering; we may use deferred splitting. By changing the split strategy, the DR-tree will achieve the maximum utilization needed.

R-trees’ efficiency depends on how effective the algorithm is, clustering data rectangles into a node. We used space-filling curves (or fractals) here, and precisely the Hilbert curve to enforce a linear ordering on data rectangles. Exactly once, a space-filling curve visits all points in a *k*-dimensional grid and never crosses. To derive an order curve *i* and the order curve *i* − 1, which can be rotated and/or mirrored accordingly, each vertex of the simple curve is substituted. When curve order tends to infinity, the resultant curve is a fractal with a fractal dimension 2 [5,6,7]. The main concept is to construct a tree structure that can act like an R-tree on scan and help deferred separation on insertion using the inserted data rectangle’s Hilbert value as the primary key. These objectives can be accomplished as follows: for each node *n* of our physical tree, they store (a) its cluster region and (b) the largest Hilbert size (LHV) of data rectangles belonging to the root *n* sub-tree.

DR-trees expand the R-tree index architectures where related nodes were self-organized in a synthetic balanced tree overlay centered on semantic relationships. The framework preserves R-trees’ index framework features: logarithmic search time in network size and minimal degree per node. Atomic devices connected to the device may be called *p*-nodes (real nodes shortcut). A DR-tree is a virtual framework spread over a collection of *p*-nodes. Terms related to the DR-tree have the prefix: “*v*-.” Thus, DR-trees nodes are called *v*-nodes (virtual network shortcut). The key points in the DR-tree composition are join/leave procedures. When a *p*-node connects, it generates a *v*-leaf. Then another *p*-node is contacted to inject the *v*-leaf into the current DR-tree. Any *v*-nodes can break during this insertion, see Algorithm 1 [35].
**Algorithm 1:** DR-tree splitting**void** onSplit (*n*:*V*_Node) 2:**If**
*n* is *V*_ Root( )then 3: new *V*_ Root = *n*.create *V*_Node( ) 4: *n*.*v*_ father = new *V*_Root 5: **End If** 6: *m* = selectChildIn (*n*.*v*_children) 7: new V Node = *m*.create *V*_Node( ) 8: *n*.*v*_children, new *V*_Node.*v*_children = divide (*n*.*v*_children) 9: new *V*_ Node.*v*_ father = *n*.*v*_father

### 3.2. Entropy Estimation

Given the conceptual (logic) DR-tree, this stage aims to eliminate unnecessary nodes by its entropy calculation. Entropy is often used as an evaluation metric that represents the consistency of a scheduling choice based on ambiguous state-of-service capacity information [36]. This approach is to omit the domain block (node) with high entropy from the domain pool. Thus, all useless domains would be eliminated from the pool, achieving a more efficient domain pool. This would minimize network overhead by decreasing the number of search nodes and improve grid computing system efficiency. The grid manager at the GCS estimation step will initialize Algorithm 1 by choosing some parameter ξ and then Algorithm 2 will be executed. Herein, the stability of the logical DR-tree relies on the ξ value for each node; these values are empirically determined (trial and error). Stability here means that the node will not be failed. As ξ increases, the uncertain information of the node increases and Quality of Service (QoS) will decrease.
**Algorithm 2:** Entropy Estimation1: Step 1: Initialization chosen parameter ξ; 2: Divide the input grid into *n*:*V*_Node 3: For (*j* = 1; *j* ≤ *n*; *j*++) { 4: *H* = entropy (*V*_Node); 5: If (*H*≤) 6: Step 2: execute Algorithm 1

### 3.3. Fault Tolerance

Given the reduced version of the logical nodes from the previous step, fault tolerance for each path that is constituted from the combination of all nodes will be estimated depending on the typical DR-tree performance [13]. If a *p*-node fails, all the sub-trees are substituted in a non-default configuration (*p*-node by *p*-node) to maintain an invariant DR-tree. The proposed model maintains fault tolerance and retains the DR-tree architecture with its invariants utilizing non-leaf *v*-node replication. A fault-tolerant solution that utilizes non-leaf node replication preserves tree connectivity when crashes arise. The pattern for replication of *v*-nodes is: *p*-root has no replica, and each *p*-node has a replica of its top *v*-node’s *v*-father.

(a) *Fault Tolerance Estimation*

Replicas are generated at join operations and updated to join both split processes. When changing a *v*-node, its holder can alert the *p*-nodes containing its replicas. Consider an *N v*-node DR-tree with degree *m: M*. Presume that upgrading a replica costs one post (message). To calculate the cost of replica changes, determining how many *v*-nodes are updated throughout a split process is also important. Let the cost be described as the value of split-time updating reproductions. Since each *v*-node has *M*−1 replicas, each update requires one post, so it costs:

(2) $$cost_{s}=\left(m+2\right)\left(M-1\right),$$

A *p-node* associated with the system may change between 0 and $\left\lfloor log_{m}\left(N\right)\right\rfloor$ splits, adding its *v-leaf* to the *v-children* of another *v-node* that is denoted in the sequence of the joined *v-node*. The latter has between *m* and *M v-children*; $\left\lceil log_{m}\left(N\right)-1\right\rceil$ *v-ancestors*; between *m* − 1 and *M* − 1 replicas. A *v-node* can have *m* to *M v-children* and therefore has *M* − *m* + 1 possible *v-children*. The split will happen only when it has exactly *M v-children*. The probability for a *v-node* to split is *p*, where:

(3) $$p=\frac{1}{M-m+1},$$

The likelihood of a *p*-node producing *k* splits is that the corresponding *v*-node and its *k*-1 first *v*-ancestor would have practically *M v*-children, although their *k*-th ancestor would not split, therefore:

(4) $$p_{k}=p^{k}\left(1-p\right).$$

Let $cost_{r}$ denote the estimated price of updating replicas when associating a *p*-node with a DR-tree:

(5) $$cost_{r}=p_{o}\left(M-1\right)+\overset{\left\lfloor \log_{m}\left(N\right)\right\rfloor }{\underset{k=1}{\sum }}(p_{k}k\;cost_{s}),$$

The first term describes the case in which no splits are made, i.e., *M* − 1 replicas of the merged *v*-node must be modified, whereas the second corresponds to the other cases. The proposed approach should have defined the case where the *v*-root splits, for a specific possibility, since it has *M* − 1 *v*-children. Even so, for *m* > 2, this likelihood is lower than *p*, so an upper limit is believed to simplify estimation.

(b) *Migration Controller*

Reinsertion and replica policy, as in [12], was used to test DR-tree integration operations utilizing internal *v*-nodes. Once a non-leaf *p*-node crash has been created by each DR-tree, the cost of device reconstruction in terms of the number of messages and the stabilization period is determined depending on both reinsertion as well as replication policies.

(1) Stabilization time: the reinsertion mechanism stagnates the system in several cycles and also at the crashed *p*-node. As stability time is the longest reinsertion time, it is proportional to log_m_(*N*).
(2) Message recovery cost: calculated as the percentage of messages required to stabilize a non-leaf *p*-node collapse. Costs range in size, but with the reinsertion policy, the amount of message propagation is much distorted, resulting in a large standard deviation.

### 3.4. Load Balance

As mentioned in [11], each SM collects every PE that enters or leaves the grid system and then communicates it to its parent LGM. The above means an input layer only needs communication when it joins or leaves its site. The device burden between computing components can be matched by utilizing the gathered information to effectively use all device resources to reduce response time for user jobs. This strategy would boost connectivity and device efficiency by minimizing overhead contact requiring capturing system details before making a task scheduling decision. The process measures for the GCS model to formalize load balancing policy are defined as:

1. Job: all jobs in the system will be represented by a job ID, job size in bytes, and a number of job instructions.
2. Processing element capacity (*PEC_ij_*): defined as the number of jobs that can be processed by the *j*th PE at full workload in the *i*th site per second. The *PEC* can be measured assuming an average number of job instructions using the PE’s CPU speed.
3. Site processing capacity (*SPC_i_*): defined as the number of jobs that can be processed by the *i*th site per second. Hence, the *SPC_i_* can be measured by summing all the PECs for all the PEs managed the *i*th site.
4. Local grid manager processing capacity (*LPC*): defined as the number of jobs that can be processed by the LGM per second. The *LPC* can be measured by summing all the *SPCs* for all the sites managed by that LGM.

(a) Load Balance Estimation

As described in [11], the load balancing strategy is multi-level and can be clarified, as follows, at each grid design level:

1. Load balance 0 (central grid manager): as stated earlier, in terms of processing capability SPCs, the LGM retains information regarding all its responsible SMs. *LPC* is the total LGM processing capacity measured as the sum of all SPCs for all LGM sites. Based on each site’s overall computing power, the LGM scheduler balances the workload of all site community members (SMs). Where *N_j_* specifies the number of jobs at an LGM in a steady state, *i*th site workload (*S_i_WL*) is its number of jobs to be delegated to the site manager and is measured as follows:
  (6) $$S_{i}WL=N_{j}\times \frac{SPC_{i}}{LPC}$$
2. Load balancing at level 1 (site manager): each SM has PEC information on all input nodes in its pool. The overall site processing capability SPC is measured by the sum of all PECs of all processing elements in the site. Where *M_j_* is specified as the number of jobs in a steady state at an SM, the SM scheduler uses the same strategy used by the LGM scheduler to distribute the load. Based on their processing power, the strategy of sharing site workload among the PE community would optimize the productivity of each PE and also boost their resource usage. On the other hand, the amount of jobs for the *i*th PE is specified as the *i*th PE workflow (*PE_i_WL*), calculated as follows:
  (7) $$PE_{i}WL=M_{j}\times \frac{PEC_{i}}{SPC}$$

(b)  Self-Stabilizing Controller

To calculate the mean job response time, one LGM scenario has been assumed to streamline the grid model. This scenario focuses on the time spent in processing elements by a job. Algorithm 3 is used to measure the traffic volume and the estimated mean response time.
**Algorithm 3:** Expected mean job response time1: Obtain *λ*, *μ* where, *λ* is the external job arrival rate from grid clients to the LGM, *μ* is the LGM processing capacity. 2: Calculate *ρ = λ/μ* as the system traffic intensity. For the system to be stable, *ρ*
must be less than 1. 3: **For**
*i* = 1 to *m* 4: Calculate *λ_i_, μ_i_* where *λ_i_* is the job flow rate from the LGM to the *i*th SM, which is managed by that LGM, *μ_i_* is processing capacity of the *i*th SM. 5: Calculate *ρ_i_ = λ_i_/μ_i_* that is the traffic intensity of the *i*th SM. 6: **For**
*j* = 1 to *n*
7: Calculate *λ_ij_, μ_ij_* where *λ_ij_* is the job flow rate from the *i*th SM to the *j*th PE managed by that SM, *μ_ij_* is the processing capacity of the *j*th PE that is managed by the *i*th SM. 8: Calculate *ρ_ij_ = λ_ij_/μ_ij_* that is traffic intensity of the *j*th PE that is managed by *i*th SM. 9: Calculate the expected mean job response time, $\;E\left[T_{g}\right]$. 10: **End for** 11: **End for**

The jobs arrive sequentially from clients to the LGM with the expectation of conforming to a time-invariant binomial mechanism, whereas inter-arrival times are independently, identically, and exponentially distributed with the arrival time of jobs/s, excluding simultaneous arrivals. An M/M/1 queue models each PE in the following website pool. Jobs that arrive at the LGM are immediately spread on the LGM structured pages with routing likelihood:

(8) $$PrS_{i}=\frac{SPC_{i}}{LPC},$$

Following the load balancing strategy (LBP), where *i* the site number

(9) $$\lambda_{i}=\lambda \times PrS_{i}=\lambda \times \frac{SPC_{i}}{LPC}.$$

Under the same scenario, the site *i* arrivals would also be automatically spread with routing likelihood on the PEs arranged by that site.

(10) $$PrE_{ij}=\frac{PEC_{ij}}{SPC_{i}},$$

Based on the LBP, in which *j* is the PE, and *i* is the site number

(11) $$\lambda_{ij}=\lambda_{i}\times PrE_{ij}=\lambda_{i}\times \frac{PEC_{ij}}{SPC_{i}}$$

As LGM arrivals are simulated to obey a Poisson process, PE arrivals would also obey a Poisson distribution. Assume that service times only at the *j*th PE in the *i*th SM are spread exponentially with fixed service rate *μ_ij_* jobs/s and reflect the processing capacity of the PE (PEC) in the high availability policy. Service management is a first-come service.

To calculate the expected mean job response time, let $E\left[T_{g}\right]$ denote the mean time spent by a job at the grid to the arrival rate *λ* and $E\left[N_{g}\right]$ denote the number of jobs in the system. Hence, the mean time spent by a job at the grid will be:

(12) $$E\left[N_{g}\right]=\lambda \times E\left[T_{g}\right]$$

$E\left[N_{g}\right]$ can be determined by averaging the mean amount of jobs at all grid sites in each PE.

(13) $$E\left[N_{g}\right]=\overset{m}{\underset{i=1}{\sum }}\overset{n}{\underset{j=1}{\sum }}E\left[N_{PE}^{ij}\right]$$

where *i* = 1, 2, *…*, *m* is the number of site managers handled by an LGM, *j* = 1, 2, *…*, *n* is the number of processing elements handled by an SM and $E\left[N_{PE}^{ij}\right]$ is the mean number of jobs in a processing element number *j* at site number *i.* As every PE is modeled as an *M/M/1* queue, $E\left[N_{PE}^{ij}\right]=\frac{\rho_{ij}}{1-\rho_{ij}}$ where

(14) $$\rho_{ij}=\frac{\lambda_{ij}}{\mu_{ij}}$$

$\mu_{ij}=\;PEC_{ij}$ for PE number *j* at site number *i*. From Equation (12), the expected mean job response time is given by:

(15) $$E\left[T_{g}\right]=\frac{1}{\lambda }\times E\left[N_{g}\right]=\frac{1}{\lambda }\times \sum_{i=1}^{m}\sum_{j=1}^{n}E\left[N_{PE}^{ij}\right],$$

Notice that the stability state *PE_ij_* is *ρ <* 1.

### 3.5. Threshold Device

**Figure 2:**
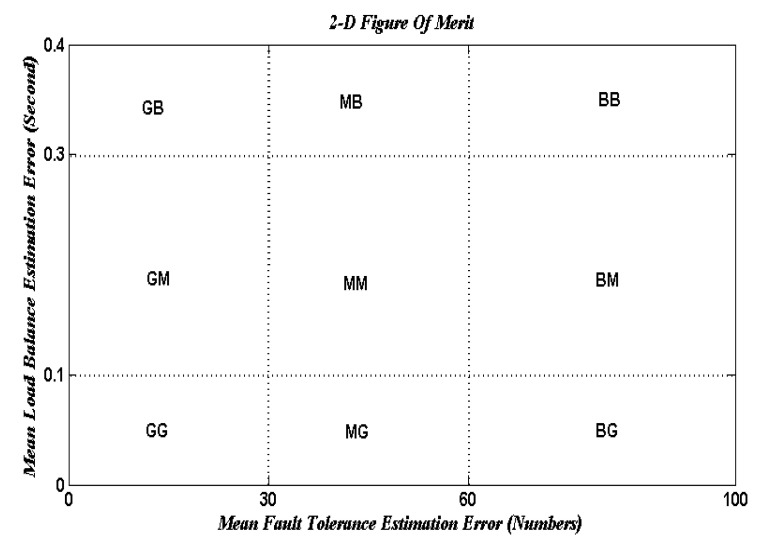
The proposed 2-D figure of merit.

The suggested approach uses e a novel 2-D figure of merit to test the network performance. A 2-D figure of merit can be seen in Figure 2. It divides the load balance (LB) fault tolerance (FT) spaces into different development conditions as follows:

The FT estimation varies between three threshold intervals (FT error units are in numbers depending on the grid size) as follows (experimentally determined):

(1) From [0 … 30] specifies a good system.
(2) From [30 … 60] specifies a medium system.
(3) The bad system is above 60.

The LB estimate ranges between three threshold periods (LB error units based on the mean response time) as follows (experimental data determined):

(1) [0 … 0.1] indicates a good system.
(2) [0.1 … 0.3] indicates a medium system.
(3) The based device over 0.3. Figure 2 can be divided into nine areas to observe the intervals discussed:
  ▪ GG: this interval is good for both FT and LB estimation.
  ▪ GM: this interval is good for FT estimation and medium for LB estimation.
  ▪ GB: this interval is good for FT estimation and bad for LB estimation.
  ▪ MG: this interval is medium for FT estimation and good for LB estimation.
  ▪ MM: this interval is medium for both FT and LB estimation.
  ▪ MB: this interval is medium for FT estimation and bad for LB estimation.
  ▪ BG: this interval is bad for FT estimation and good for LB estimation.
  ▪ BM: this interval is bad for FT estimation and medium for LB estimation.
  ▪ BB: this interval is bad for both FT and LB estimation.

Finally, to improve the calculation of fault tolerance, replication time and message cost must be minimized, which would raise the possibility of completed work. On the other hand, mean job response time would be reduced to boost the load balancing measurement and this will cause an improvement in the number of jobs/s. A novel two-dimension figure of merit is suggested to describe the network effects on load balance and fault tolerance estimation. The suggested model would be improved by using optimization techniques to approximate the optimum replication time value and mean job response time to achieve a GG framework in the 2-D figure of merit. Three separate optimization methods are used to achieve the optimal approach, namely: genetic algorithm, ant colony optimization, and particle swarm optimization (GA, ACO, and PSO). The aim is to compare these three optimization methods. In the first step, each user submits their computing jobs to the GCS with their hardware specifications. The GCS answers the user by sending the results after completing the job processing. This model follows the same measures as model one, but it includes extra modules called “optimization strategies” that take their input from the entropy estimation module (logical network) and send their output to the fault tolerance and load balancing estimation modules (outputs replication time and mean job response time, respectively).

## 4. Results

All experiments were conducted based on a dataset that was collected from http://strehl.com/. A sample of 500 records is generated for the node’s entropy measurement according to five distributions: random, exponential, normal, uniform, and Poisson. All of these measurements follow Gaussian clusters with means of (−0.227, 0.077) and (0.095, 0.323) and an equal variance of 0.1. Table 1 illustrates a sample of the records and attributes of each node that includes queue size, task time, CPU speed, and memory size. The simulation model was implemented on: CPU processor: Intel (R) Core (TM) i3-243M CPU@2.40 GHz 2.40 GHz. RAM: 4.00 GB. System type: 64-bit operating system. Operating system: Microsoft Windows 7 Professional. A simulation model is constructed using a MATLAB simulator to assess the performance of the grid computing model. This simulation model consists of one local grid manager who manages several site managers. The statistics MATLAB toolbox is utilized to compute the entropy.

The accuracy of the proposed model was evaluated by four well-known measures [37]: load balance estimation: this measure is used to evaluate the mean job response time, and this denotes the period spent on the grid by varying arrival times. The objective is to decrease mean job response time and this will cause an increase in the number of jobs/s. Fault tolerance estimation: this measure is used to evaluate the replication cost of the DR-tree; this could be assessed by the summation of the probability of not splitting for every virtual node and its update replica message cost. The objective is to decrease the replication cost. Gain: to evaluate the improvement ratio for the load balance with respect to the traditional load balance model, which can be calculated by gain = (traditional mean job response time–planned mean job response time)/traditional mean job response time. Finally, system utilization: to evaluate the number of required resources with respect to grid size. The objective is to decrease the grid size and consequently decrease the number of resources.

### 4.1. Experiment One: Test the Performance of the Proposed Model in Terms of Load Balancing

Objective: To validate the benefits of implementing the proposed DR-tree model for grid computing networks; this experiment compares it with related load balancing algorithms discussed in [11]. The aim is to decrease the mean job response time and consequently increase the number of jobs/s.

**Figure 3:**
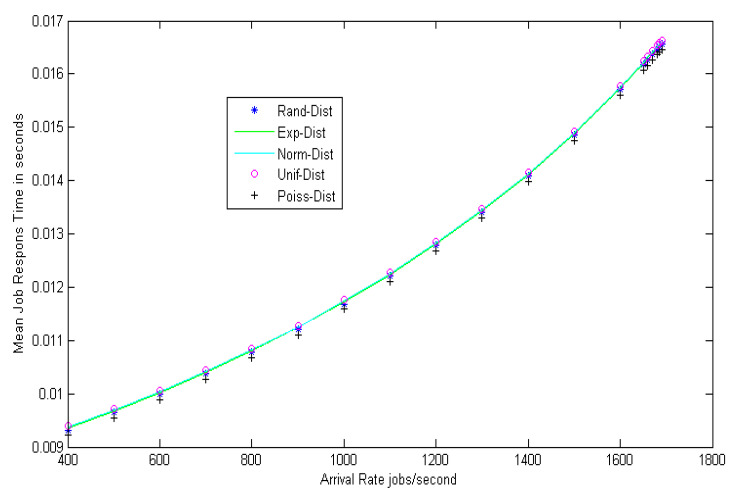
Load balance estimation.

**Figure 4:**
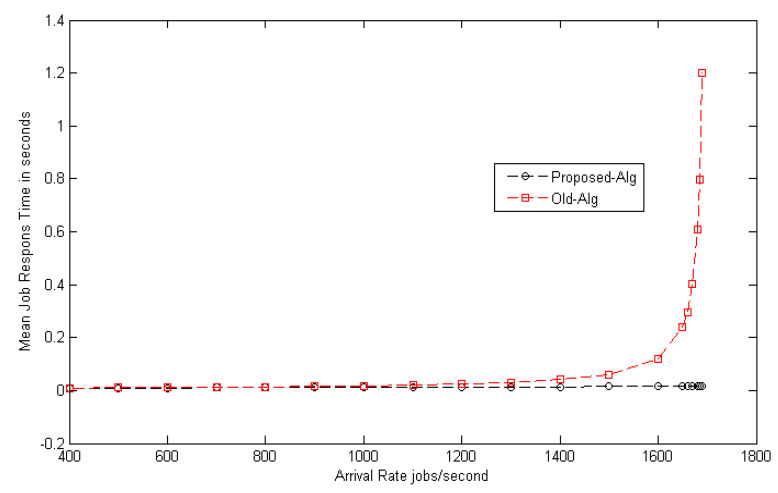
Load balance estimation with the traditional algorithm.

Observation: Figure 3 shows the load balance of the grid network at different arrival rates. The mean job response time for different random distributions, such as random (Rand-Dist), exponential (Exp-Dist), normal (Norm-Dist), uniform (Unif-Dist), and Poisson (Poiss-Dist) are calculated and, after many trials, it can be shown that the same results were obtained for all distributions. The results confirm that the mean job response time increases approximately in a linear way as the arrival rate increases. Figure 4 shows the comparison between the load balance of the proposed model and the traditional algorithm mentioned in [11]. The results from Figure 4 prove the superiority of the proposed model. The suggested model improves the mean job response time by decreasing it with a ratio of 26% (gain) as compared to the traditional one. Additionally, it can be observed from the table that the different distributions of mean job response times do not affect the stability of the model.

**Figure 5:**
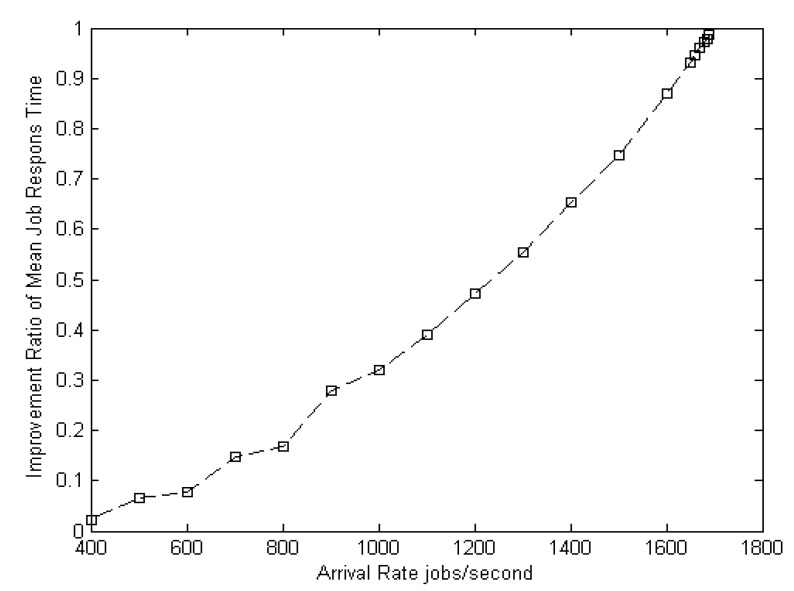
Load balance improvement ratio.

Figure 5 shows the improvement ratio of the suggested model as a function of the arrival rates. The suggested model improves the load balance by 98% from a configuration with an arrival rate of 400 jobs/s to a configuration with an arrival rate of 1690 jobs/s in the different distributions.

Discussion: One possible explanation of these results is that the load balancing achieved by the proposed model is asymptotically optimal because its saturation point intensity ≈ 1 is very close to the saturation level of the grid computing model [11]. Furthermore, the suggested model is more stable at different arrival rate distributions because of the utilized DR-tree properties that mainly depend on the reduction version of the tree. This reduction version of the grid network tree mainly improves the load balancing policy as compared to the alternative model in [11] that depend on the whole grid network tree. Moreover, within the suggested model, the information of any processing element joining or leaving the grid system is collected at the associated site manager which in turn transmits it to its parent local grid manager. This means that communication is needed only if a processing element joins or leaves its site. All of the collected information is used in balancing the system workload between the processing elements to efficiently utilize the whole system resources, aiming to minimize user job response time. This policy minimizes the communication overhead involved in capturing system information before making a load balancing decision that improves the system performance.

### 4.2. Experiment Two: Test the Performance of the Proposed Model in Terms of Fault Tolerance

Objective: The second set of experiments was conducted to confirm the efficiency of the suggested DR-tree-based model in terms of replication time and message cost for grid computing networks; the aim is to decrease replication time and message cost and this will cause an increase in the probability of a job being completed. In general, after building the logical grid computing model and executing the load balancing stage, the grid network size influences the time taken for jobs to be completed. So, if the grid size of the network is minimized, the probability of the job being completed is maximized.

**Figure 6:**
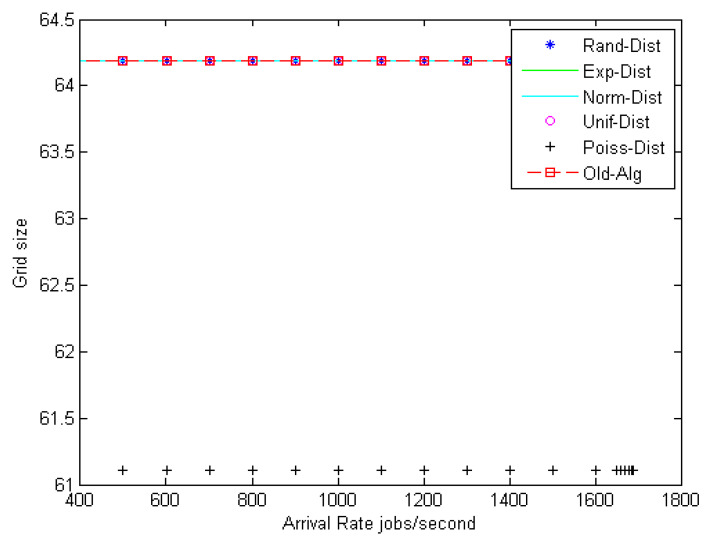
Fault tolerance estimation (grid size with different arrival rates).

**Figure 7:**
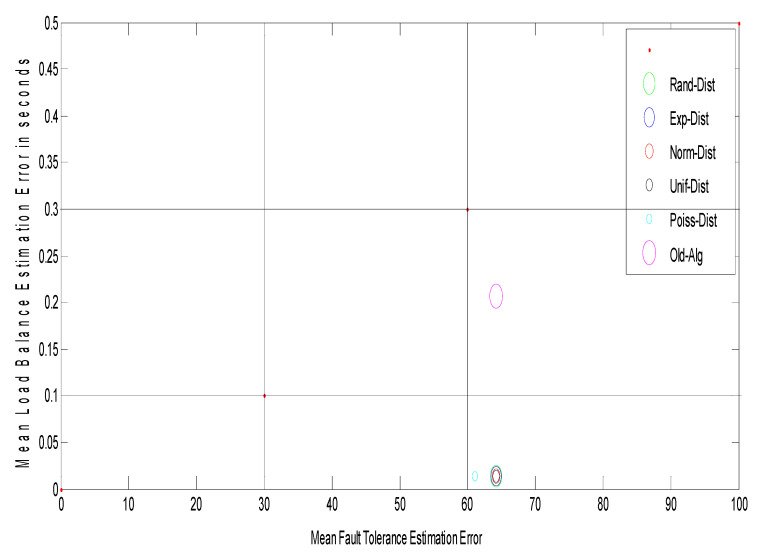
Path selectors with 2-D figure of merit.

Observation: Figure 6 shows the relationship between grid size and the arrival rates for different random distributions, such as random (Rand-Dist), exponential (Exp-Dist), normal (Norm-Dist), uniform (Unif-Dist), and Poisson (Poiss-Dist). The results reveal that for different arrival rates, the Poisson distribution yields a minimum grid size as compared with other distributions. It has an improvement ratio of about 4.6%. Furthermore, Figure 7 shows a 2-D figure of merits that depicts the relationship between mean load balance estimation error and mean fault tolerance estimation error. It can be inferred that the Poisson distribution gives the best stability system compared to all different distributions and the job response time algorithm. As shown in Figure 7, given the best result for the Poisson distribution, the load balance is still the same, and this confirms the stability condition of the DR-tree model. The next step tries to enhance load balance, and the fault tolerance of the suggested model depends on different entropy values.

**Figure 8:**
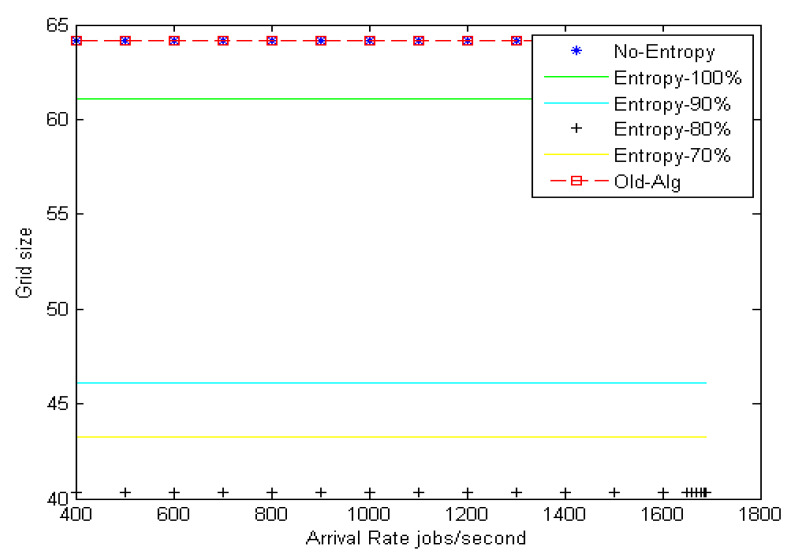
Fault tolerance estimation with different entropy for Poisson distribution.

**Figure 9:**
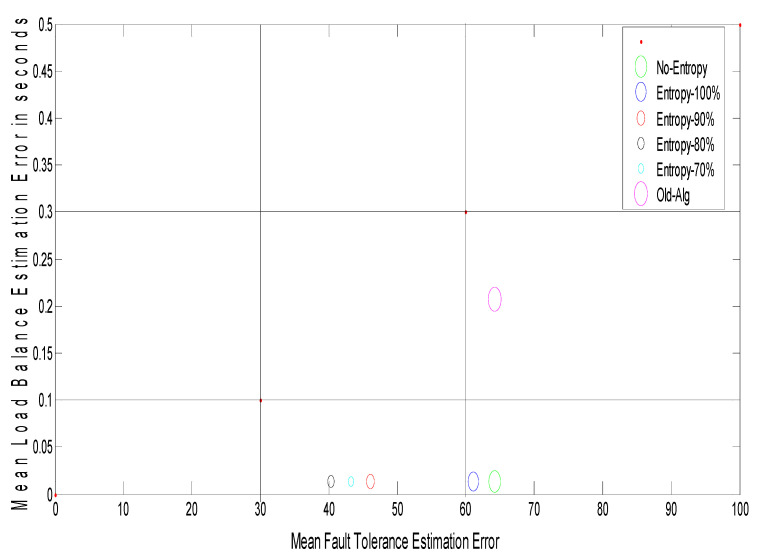
Path selectors with 2-D figure of merit with different entropy for Poisson distribution.

Figure 8 shows the grid size at different arrival rates for the Poisson distribution with different entropy threshold values; the improvement ratio for fault tolerance in terms of grid size is 33% at an entropy value of 80%. Figure 9 shows the system stability in terms of load balance and fault tolerance estimation errors after enhancement. The results reveal that when decreasing the entropy threshold value ε to less than 80%, the stability of the system decreases. The suggested model yields an improvement ratio of 98% for load balance and 33% for fault tolerance as compared with initial conditions.

Discussion: As the proposed model depends on the entropy-based DR-tree index structure through which the number of nodes that have properties depends on reducing the error estimation for each of load balance and fault tolerance, these selected nodes represent the logical structure and reduce the network size and reducing the network size leads, accordingly, to reducing the mean job response time and replication time.

### 4.3. Experiment Three: Test the Performance of the Proposed Model in Terms of System Utilization

Objective: To measure the optimum solution for the resources available within the grid network, this set of experiments was conducted to illustrate the effect of three different optimization techniques to minimize the number of resources with the grid. The aim is to decrease the number of resources with respect to grid size to enhance grid utilization. Table 2 illustrates the parameters for factors and levels of setup of different optimization techniques. The error estimation parameter value ε represents the fitness functions.

**Figure 10:**
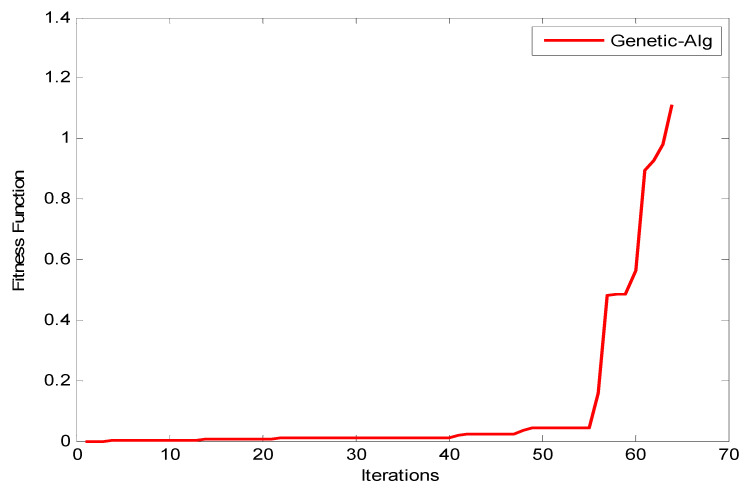
Fitness function of GA.

**Figure 11:**
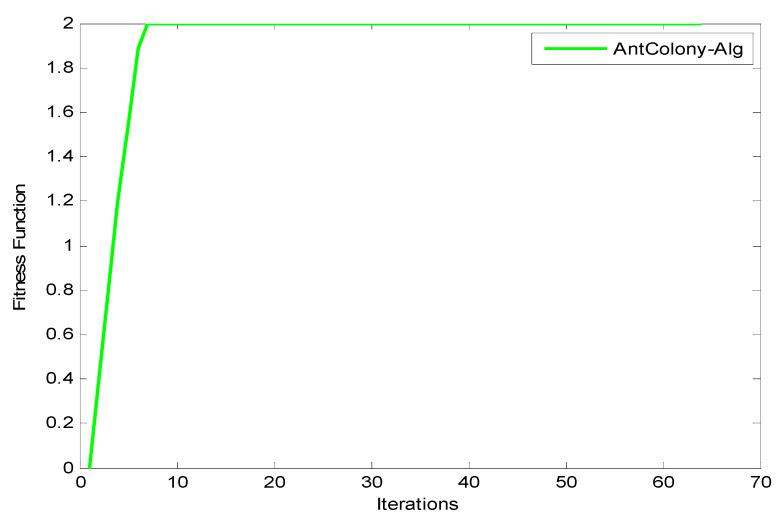
Fitness function of ACO.

**Figure 12:**
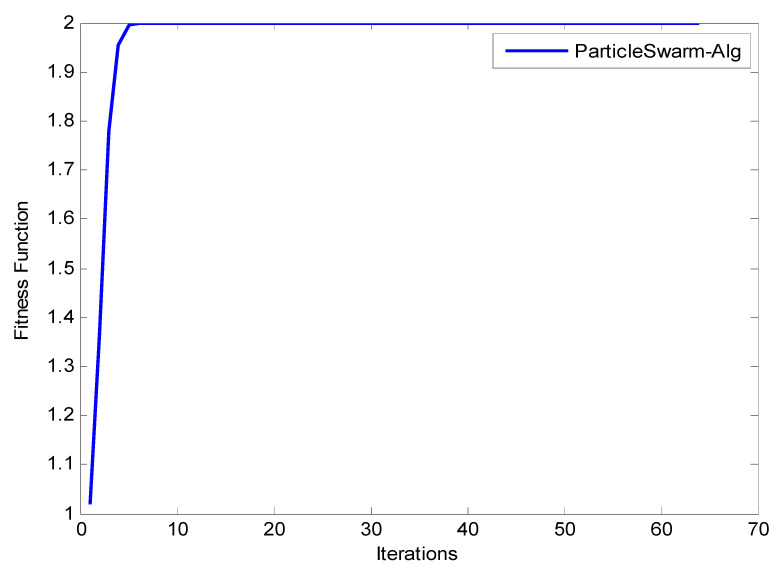
Fitness function of PSO.

**Figure 13:**
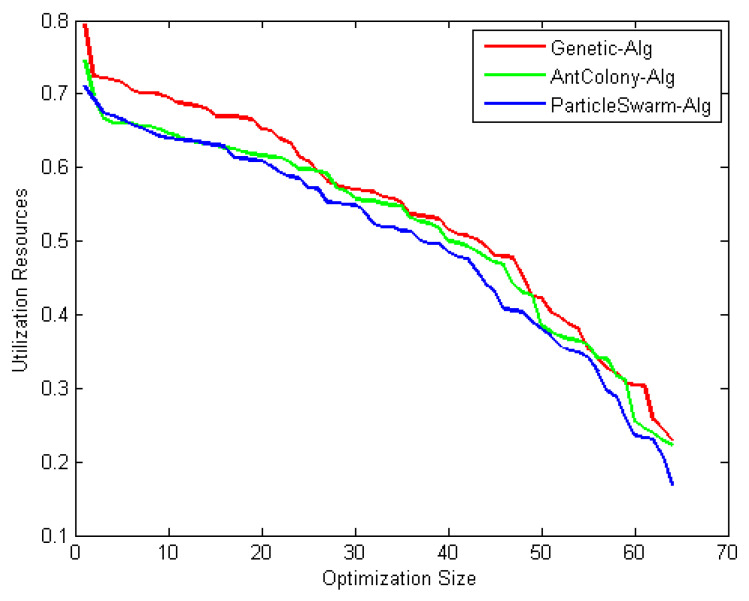
System utilization, comparative study.

Observation: The results shown in Figure 10, Figure 11 and Figure 12 reveal that as the number of iterations increases, the ξ value increases for GA, ACO, and PSO, respectively. The increasing of the curve tends to become stable approximately after the 8th iteration for both PSO and ACO, while GA reaches stability at the final iteration to obtain an optimal value. It can also be observed that the PSO optimization technique yields the best ξ value as it reaches a stable value faster than ACO. Figure 13 shows the system utilization resources as a function of optimal size (number of iterations for each optimization technique). As the optimization size increases, the system utilization decreases. When comparing the three optimization algorithms with respect to system utilization, the results confirm that they almost give the same results (about 75%) but it could be shown that the PSO algorithm gives the best performance.

Discussion: Several conclusions were derived from the results: (i) in terms of effectiveness, the three algorithms performed equally well to search for the optimal solution. (ii) In terms of efficiency, PSO is the fastest among the three algorithms to find the optimum result, followed by ACO and then GA. (iii) In terms of consistency, all methods are proved to be consistent in solving this construction site layout problem. This study contributes to the decision in determining an appropriate solution algorithm for the construction site layout problem. The population size in the parameter design gives a significant effect on the objective solution. All methods performed equally well in terms of effectiveness. However, PSO appears to achieve the minimum mean of cost as compared to GA and ACO. This is due to the implementation of the craziness concept in the PSO mechanism; i.e., advantages of randomly reinitializing particles, a process referred to as craziness. PSO is shown to be the fastest algorithm that converges on the minimum cost. For consistency, all methods are proved to be consistent in finding solutions for both cases. This is due to the diversification components that prevent the algorithm from becoming trapped in local optima and are able to explore the solution in search space until they finally converge on the best objectives.

The experimental results that were found through a simulator confirmed that the suggested model can be improved by up to 98% for load balance from the initial condition; furthermore, it outperforms the related work by an average of 26% for job response time and 33% for fault tolerance. Furthermore, by utilizing different optimization algorithms for finding the optimal number of resources (system utilization), the suggested model decreases the number of resources by an average of 75%. In general, fault-tolerant load balancing is a critical issue for the efficient operation of grid computing environments in distributing the jobs. These results show that passive replication has been combined with distributed load balancing in the grid and suggest a new way to control the stability of the grid networks. Message exchanges between resources in this model are simple and small, thereby preventing network congestion even during heavy job arrival rates. This model integrates static and dynamic load balancing techniques to locate effective sites, identifies system imbalance in the shortest time when any site becomes ineffective, and fills the imbalance with a new site.

## 5. Conclusions

In a grid environment, many researchers have proposed various scheduling algorithms for improving the performance of the grid system. This paper began by studying and understanding several aspects of grid computing. In the literature survey, various algorithms and methods were identified and studied. Even though many researchers proposed various scheduling algorithms, it is found that there is no efficient and effective scheduling algorithm that gives a combined solution for many issues. This research proposed efficient and effective scheduling algorithms in which various issues, such as user satisfaction, load balancing, and fault tolerance, are addressed by considering the parameters such as failure rate, load state, user deadline, and resource utilization of the resources for scheduling.

In general, the main contributions of this work can be highlighted as follows: (1) proposing a new adaptive model to improve fault tolerance and load balancing for the grid computing environment. This model depends on an advanced fractal transform that enhances the tree model structure of the grid computing environment to enhance the network performance parameters affected by fault tolerance and load balance equally. An estimate of the fault tolerance and load balance for the network parameters was calculated based on a fractal transform. (2) The grid computing routing protocol is enhanced by improving fault tolerance with load balance estimation in a novel 2-D figure of merit. The improvement of the fault tolerance estimation is carried out by reducing replication time and message cost and this results in an increase in the probability of job completion. On the other hand, reducing mean job response time results in an enhancement of the load balance estimation, and this in turn induces an increase in the number of jobs/s. The experimental results that were found through a simulator confirmed that the suggested models can be improved by up to 98% for the system and outperform the related work by an average of 26% for job arrival rate and 33% for fault tolerance. Furthermore, by utilizing different optimization algorithms for finding the optimal number of resources (system utilization), the suggested model decreases the number of resources by an average of 75%.

Future work may include: (1) applying the proposed model to a real-time environment. (2) The security of the proposed work has not been considered, therefore, researchers may study the security aspects of this work. (3) Some other user requirements, such as cost for execution, may be considered. In addition to that, other passive failure handling mechanisms, such as checkpointing, may be considered. (4) The jobs may arrive in a random manner. So, the dynamicity of the jobs may be considered for testing the suggested models. (5) The proposed model is tested with 64 resources and a varied number of jobs of up to 1000. In the future, the number of resources and jobs may be increased and tested as an extension of the proposed models. (6) Exploring modeling other characteristics such as input/output (I/O) behavior, memory access pattern, cache effects, and seeking to build corresponding scheduling strategies that utilize these parameters to form efficient scheduling strategies.

## Figures and Tables

**Table 1:** Sample of node attributes.

Node_id	Q_Size	Task Time (sec)	CPU Speed (GHz)	Memory Size (GB)
1	90.73618432	453.0043176	67.84965223	84.77457651
2	95.28959685	689.9301012	124.7546078	62.6737682
3	56.34934081	254.068019	193.9615938	59.47512475
4	95.66879281	735.4414792	101.057859	92.54742974
5	81.61796231	128.6495617	137.7901626	35.72551169
6	54.87702025	349.2306865	83.57179092	78.14802062
7	63.92491094	141.5542516	162.6900589	77.83561849
8	77.34407596	187.4186031	88.26426732	44.24012623
9	97.87534177	841.1120455	125.8935577	61.10394767
10	98.24442676	725.3457607	154.8615084	16.82688606
11	57.88065408	385.3895321	183.6354879	14.85551068
12	98.52963909	955.199844	193.8937138	57.77177977
13	97.85834741	131.0014725	132.0823295	80.12505071
14	74.26878244	494.8699237	70.79366642	94.06096158
15	90.01402344	443.4026114	72.39410083	21.69155876
16	57.09431693	788.9651093	88.62623812	61.19412948
17	71.08806413	815.679911	176.1075884	52.2451577
18	95.78677626	268.1853441	88.14232685	11.07118626
19	89.61036648	540.7879562	172.1427239	40.341038
20	97.97462132	501.0275806	86.52874531	24.59640774
21	82.78703496	681.6817091	189.3895435	81.48560866
22	51.78558393	738.4283478	102.4975649	38.00935378
23	92.45646529	779.2180138	79.48928756	57.5679822
24	96.69966239	348.4225693	87.6625787	24.90838565
25	83.93675774	711.7324092	142.4067014	64.17837473
26	87.88700653	689.5882036	120.9933273	33.66741561
27	87.15662341	246.3505617	102.7489261	68.86711886

**Table 2:** Optimization techniques setup.

Factors	Minimum Level	Maximum Level
Genetic Algorithm (GA)		
Number of chromosomes	32	64
Number of iterations	500	1000
Cross-mutation rate	0.4	0.8
Mutation rate	0.01	0.02
Ant Colony Optimization (ACO)		
Number of ants	32	64
Number of iterations	500	1000
Weight of pheromone (α)	1	2
Evaporation rate (λ)	0.4	0.8
Particle Swarm Optimization (PSO)		
Number of particles	32	64
Number of iterations	500	1000
Acceleration constant (*C*_1_ = *C*_2_)	2	4
Inertia weight (*W*)	0	1

## References

[1] Chetan E., Sharma E.D. (2015). Fractal image compression using quad tree decomposition & DWT. Int. J. Sci. Eng. Res..

[2] Jahromi M.N., Falahati A., Edwards R.M. (2011). Application of fractal binary tree slot to design and construct a dual band-notch CPW-ground-fed ultra-wide band antenna. IET Microw. Antennas Propag..

[3] Kasheff Z., Walsh L., Esmet J., Prohaska R., Inventors, PERCONA LLC, Assignee (2015). Replication in a NoSQL System Using Fractal Tree Indexes. U.S. Patent.

[4] Iano Y., da Silva F.S., Cruz A.M. (2005). A fast and efficient hybrid fractal-wavelet image coder. IEEE Trans. Image Process..

[5] Sharma M., Pachori R.B., Acharya U.R. (2017). A new approach to characterize epileptic seizures using analytic time-frequency flexible wavelet transform and fractal dimension. Pattern Recognit. Lett..

[6] Chaudhari R.E., Dhok S.B. (2012). Review of fractal transform based image and video compression. Int. J. Comput. Appl..

[7] Dai M., Chen Y., Wang X., Sun Y., Su W. (2018). Spectral analysis for weighted tree-like fractals. Phys. A Stat. Mech. Appl..

[8] Berman F., Fox G., Hey A. (2003). Grid Computing: Making the Global Infrastructure a Reality, Wiley Series in Communications Networking & Distributed Systems.

[9] Elavarasi S., Akilandeswari J., Sathiyabhama B. (2011). A Survey on Partition Clustering Algorithms. Int. J. Enterp. Comput. Bus. Syst..

[10] Sharma R., Soni V.K., Mishra M.K., Bhuyan P. (2010). A survey of job scheduling and resource management in grid computing. World Acad. Sci. Eng. Technol..

[11] El-Zoghdy S.F. (2011). A load balancing policy for heterogeneous computational grids. Int. J. Comput. Sci. Appl..

[12] Valero M., Arantes L., Potop-Butucaru M., Sens P. Enhancing fault tolerance of distributed R-tree. Proceedings of the 2011 5th Latin-American Symposium on Dependable Computing.

[13] Kumar A., Yadav R.S., Ranvijay A.J. (2011). Fault tolerance in real time distributed system. Int. J. Comput. Sci. Eng..

[14] Cao J., Spooner D., Jarvis S., Nudd G. (2005). Grid Load Balancing using Intelligent Agents. Future Gener. Comput. Syst..

[15] Yagoubi B., Slimani Y. (2006). Dynamic Load Balancing Strategy for Grid Computing. World Acad. Sci. Eng. Technol..

[16] Yan K., Wang S., Wang C., Chang C. (2009). Towards A Hybrid Load Balancing Policy in Grid Computing System. J. Expert Syst. Appl..

[17] Hao Y., Liu G., Wen N. (2012). An Enhanced Load Balancing Mechanism based on Deadline Control on GridSim. Future Gener. Comput. Syst..

[18] Cao J., Spooner D., Jarvis S., Saini S., Nudd G. Agent-based Grid Load Balancing using Performance Driven Task Scheduling. Proceedings of the International Parallel and Distributed Processing Symposium.

[19] Cao J. Self-Organizing Agents for Grid Load Balancing. Proceedings of the 5th IEEE/ACM International Workshop on Grid Computing.

[20] Balasangameshwara J., Raju N. (2012). A Hybrid Policy for Fault Tolerant Load Balancing in Grid Computing Environments. J. Netw. omput. Appl..

[21] Balasangameshwara J., Raju N. A Fault Tolerance Optimal Neighbor Load Balancing Algorithm for Grid Environment. Proceedings of the International Conference on Computational Intelligence and Communication Systems.

[22] Ghemawat S., Gobioff H., Leung S. (2003). The Google File System. ACM Spec. Interest Group Oper. Syst. Rev..

[23] Lee K.-H., Lee Y.-J., Choi H., Chung Y.D., Moon B. (2011). Parallel Data Processing with MapReduce: A Survey. ACM Sigmod Rec..

[24] Mishra S.K., Sahoo B., Parida P.P. (2020). Load balancing in cloud computing: A big picture. J. King Saud Univ. Comput. Inf. Sci..

[25] Rathore N.K., Rawat U., Kulhari S.C. (2020). Efficient hybrid load balancing algorithm. Natl. Acad. Sci. Lett..

[26] Wided A., Okba K. (2020). A new agent based load balancing model for improving the grid performance. Multiagent Grid Syst..

[27] Matani A., Naji H.R., Motallebi H. (2020). A Fault-Tolerant Workflow Scheduling Algorithm for Grid with Near-Optimal Redundancy. J. Grid Comput..

[28] Khaldi M., Rebbah M., Meftah B., Smail O. (2020). Fault tolerance for a scientific workflow system in a cloud computing environment. Int. J. Comput. Appl..

[29] Shukla A., Kumar S., Singh H. (2020). Fault tolerance based load balancing approach for web resources in cloud environment. Int. Arab J. Inf. Technol..

[30] Patni J.C. Centralized Approach of Load Balancing in Homogenous Grid Computing Environment. Proceedings of the 3rd International Conference on Computers in Management and Business.

[31] Goswami S., Mukherjee K. (2020). High Performance Fault Tolerant Resource Scheduling in Computational Grid Environment. Int. J. Web Based Learn. Teach. Technol..

[32] Rezaeipanah A., Mojarad M., Fakhari A. (2020). Providing a new approach to increase fault tolerance in cloud computing using fuzzy logic. Int. J. Comput. Appl..

[33] Posner J., Reitz L., Fohry C. (2020). A Comparison of Application-Level Fault Tolerance Schemes for Task Pools. Future Gener. Comput. Syst..

[34] Arora M., Das S.K., Biswas R. A de-centralized scheduling and load balancing algorithm for heterogeneous grid environments. Proceedings of the International Conference on Parallel Processing.

[35] Bianchi S., Datta A., Felber P., Gradinariu M. Stabilizing Peer-to-Peer Spatial Filters. Proceedings of the 27th International Conference on Distributed Computing Systems.

[36] Hassaballah M., Makky M., Mahdy Y. (2005). A Fast Fractal Image Compression Method based Entropy. Electron. Lett. Comput. Vis. Image Anal..

[37] Rathore N., Chana I. (2014). Load Balancing and Job Migration Techniques in Grid: A Survey of Recent Trends. Wirel. Pers. Commun..

